# Natural Progression of Biochemical Markers of Biliary Tract Obstruction in Patients with Gallstone Pancreatitis

**DOI:** 10.1155/2009/820749

**Published:** 2009-05-25

**Authors:** Mikael Petrosyan, Joaquin J. Estrada, Sirius Chan, Heather Rosen, Thomas V. Berne, Rodney J. Mason

**Affiliations:** Division of Emergency (Non-Trauma) Surgery, Department of Surgery, Keck School of Medicine, University of Southern California Medical Center and Los Angeles County Hospital, Los Angeles, CA 90033, USA

## Abstract

The presenting pattern and natural progression of biochemical markers of biliary tract obstruction in patients with gallstone pancreatitis have not been elucidated. We analyzed serial values of bilirubin levels following admission to discharge in 143 patients. Ninety-four of patients demonstrated a Decrescendo (falling) pattern of bilirubin levels from admission until normalization at 21 hours (median). Forty-nine patients demonstrated a Crescendo-Decrescendo (initially rising) pattern with peak levels of bilirubin occurring at 39 hours after admission followed by a subsequent normalization after a median of 119 hours. Patients in the Decrescendo group were significantly younger (33 versus 41 years, *P* = .02) and more patients had experienced symptoms for greater than 48 hours (65% versus 47%, *P* = .05). Ten percent of patients in the Decrescendo group and 29% of patients in the Crescendo-Decrescendo group underwent ERCP (*P* = .02). Normalization of biochemical markers after ERCP was significantly delayed in both groups compared to no ERCP. Older patients present earlier, with higher bilirubin levels and normalize slower than younger patients, perhaps due to fibrosis of the ampulla and decreased compliance of the common bile duct. Patients who disobstruct spontaneously (90%) normalize quicker than patients undergoing ERCP.

## 1. Background

In Western societies gallstones are a major cause of acute pancreatitis. The passage of gallstones through the biliary tract and obstruction of the ampulla have both been associated with the development of acute pancreatitis [1–4]. Numerous studies have shown a high prevalence of common bile duct (CBD) stones or ampullary inflammation with obstruction of the biliary tree in patients operated on early after admission [1, 5–8]. By contrast, patients operated on later in the course of the illness exhibit few common bile duct stones or findings of ampullary obstruction. In conjunction with the recovery of gallstones in the feces [3, 4] of patients with gallstone pancreatitis it is felt that in most patients the CBD stones pass spontaneously and that the obstruction is usually temporary. 

We hypothesize that in gallstone pancreatitis findings of an early ampullary obstruction followed by spontaneous disobstruction as documented in the literature [1–8] should therefore be reflected in the patients' serum, with a rise and fall of bilirubin. The aim of the study was to analyze the natural progression of this biochemical marker of biliary tract obstruction in patients with gallstone pancreatitis. The purpose is to use these observations to determine the timing of spontaneous biochemical resolution and to allow for informed management decisions, such as need for ERCP, preoperative use of MRCP, and proper timing of cholecystectomy.

## 2. Patients and Methods

From November 2006 to November 2007, 207 consecutive patients with a clinical history consistent with pancreatitis were admitted to the Emergency Surgical Services (Non-Trauma) of the Los Angeles County Hospital + University of Southern California Medical Center. Patients were included in this study if they had abdominal pain typical of acute pancreatitis, radiological evidence of gallstones in the biliary system, and serum amylase and lipase levels greater than 3 times normal value (amylase >390 U/l and Lipase >180 U/l) together with elevated levels of serum bilirubin and/or alkaline phosphatase. Those patients with a history of alcohol abuse, hypercalcemia, hypertriglyceridemia, trauma, or reported use of a medication known to cause pancreatitis were excluded from the study. A total of sixty-four patients did not meet the inclusion criteria. The study population therefore consisted of 143 consecutive patients with gallstone pancreatitis. There were 27 male patients (19%) and 116 females (81%) with a median age of 35 years (19–94). The median length of hospital stay was 6 days (2–72). 

Once the diagnosis of gallstone pancreatitis had been established serial laboratory tests of liver function were performed every 6 hours until resolution of the patient's acute illness, hepatic derangement, or discharge from hospital. Since emergent ERCP is not readily available at our facility, this policy allowed us to closely monitor patients and select patients who truly needed emergent ERCP. Based on our clinical laboratory's normal range of values, abnormal values were determined as follows: total bilirubin >1 mg/dL, direct bilirubin >0.3 mg/dL, and alkaline phosphatase 140 U/l. This was an observational study, and the primary end point was to determine the natural history of bilirubin levels in patients with gallstone pancreatitis. Serial bilirubin levels were analyzed as a function of time in hours and the following secondary endpoints determined the following.

- *T*_Admission_ = the time period corresponding to the first laboratory test upon presentation to hospital.
- *T*_Peak_ = the time period with the highest values of serum bilirubin.
- Time to Peak = *T*_Peak_ − *T*_Admission_.
- *T*_Normal_ = the time period corresponding to when serum of bilirubin was consistently within normal values.
- *T*_ERCP_ = the time period corresponding to the performance of the endoscopic retrograde cholangiopancreatography (ERCP).
- Time to Normalization = *T*_Normal_ − *T*_Peak_. For patients undergoing ERCP, Time to Normalization was calculated as *T*_Normal_ − *T*_ERCP_.

We found a similarity between serum bilirubin and alkaline phosphatase whenever there was an increasing trend. There was a poor concordance between bilirubin and alkaline phosphatase with regard to Time to Normalization. This we believe is due to the long half life of alkaline phosphatase of 3 days [9]. Furthermore alkaline phosphatase is not excreted in the bile. Previous studies have also shown a good concordance with biliary disobstruction and falling levels of bilirubin [10]. We therefore chose only bilirubin levels in calculating Time to Normalization. 

 Patients were admitted and treated with a standard protocol: fluid resuscitation with Lactated Ringer's Solution, nothing per mouth, serial laboratory testing every six hours, and clinical assessment every 6 hours. The severity of pancreatitis was assessed at admission according to Ranson's criteria [11]. Three patients were classified as severe pancreatitis with 3 or more Ranson's criteria on admission and prophylactic antibiotics were started. There was no mortality in this series. 

Right upper quadrant ultrasonography was performed on admission. Evidence of ongoing biliary obstruction was assessed biochemically in conjunction with ultrasonography. Common bile duct (CBD) dilation was determined using the dimensions established by Bachar et al. [12] for the normal upper limit of CBD in patients according to age group. Dilatation was considered present if the CBD diameter of the patients was greater than the mean ± 2 standard deviations of normal subjects. The CBD could not be adequately delineated in 2 patients. 

 Patients exhibiting signs and symptoms consistent with cholangitis and those patients with persistent and/or worsening pain were evaluated for ERCP. Cholecystectomy was performed once the acute illness had resolved and bilirubin levels had normalized.

## 3. Statistical Analysis

All data is expressed as medians ± interquartile range unless otherwise stated. Paired data was analyzed using Wilcoxon sign rank test, and unmatched data using the Wilcoxon rank sum test. The two-sample *t* test was used for continuous variables. Categorical data was analyzed using the Fisher exact test. All tests were done by using a 2-sided test with significance at *P* < .05.

## 4. Results

Serial biochemical analysis of serum bilirubin and alkaline phosphatase revealed two distinct patient groups: a “Decrescendo” group demonstrated an immediate and sustained decrease in bilirubin levels following admission to hospital and a “Crescendo-Decrescendo” group demonstrated an initial rise in serum bilirubin levels to a peak value followed by a subsequent decrease. A total of 94 (66%) patients showed the Decrescendo trend, and 49 (34%) patients the Crescendo-Decrescendo trend (Table 1). There were no gender differences, but patients in the Decrescendo group were significantly younger. The duration of abdominal pain was similar between the two groups (*P* = .83). Interestingly, significantly (*P* = .05) fewer patients presented with complaints of abdominal pain prior to admission in the Decrescendo group compared to the Crescendo-Decrescendo group.

### 4.1. Bilirubin Levels on Presentation

**Figure 1:**
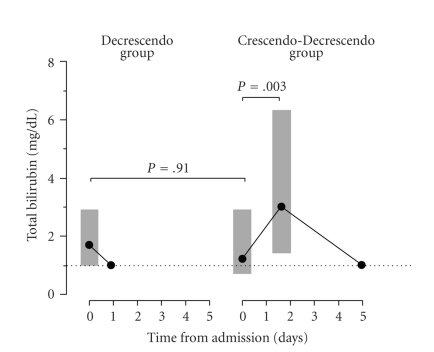
Trends of bilirubin levels during hospital stay. Data is presented as medians (solid circle) and interquartile range (grey box). Bilirubin levels on admission (Day 0) are shown for both the Decrescendo and Crescendo-Decrescendo groups. Time to peak bilirubin values are shown for Crescendo-Decrescendo Group. Median time to normalization of bilirubin levels depicted for both groups. There was no significant difference in bilirubin level on admission between those patients in the Decrescendo group compared to those in the Crescendo-Decrescendo group (*P* = .91). The peak bilirubin levels in the Crescendo-Decrescendo group of patients were significantly higher than the levels on admission (*P* = .003). Dashed (- -) line shows normal bilirubin level (1 mg/dL).

**Figure 2:**
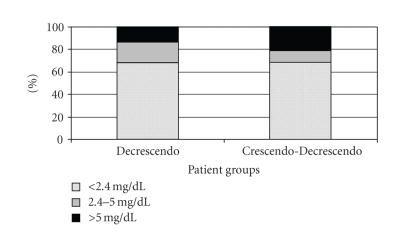
Stratification of admission bilirubin levels based on likelihood of ampullary obstruction. Bilirubin levels <2.4 mg/dL (unlikely), bilirubin levels 2.4–5 mg/dL (possible), bilirubin levels >5 mg/dL (likely). Depicted as percentage contribution of each category in each group. No differences were noted between patients in the Decrescendo group compared to the Crescendo-Decrescendo group (*P* = .25).

The median bilirubin level in the Decrescendo group on presentation (1.7 ± 1.9 mg/dL) was similar (*P* = .91) to the median bilirubin in the Crescendo-Decrescendo group (1.2 ± 2.2 mg/dL) (Figure 1). The peak bilirubin level occurred after a median of 39 ± 74 hours in the Crescendo-Decrescendo group. The peak value (3.0 ± 4.9 mg/dL) was significantly higher than the initial value at presentation (*P* = .003). Stratification of bilirubin levels on admission into those patients with obvious (>5 mg/dL), possible (2.4–5 mg/dL), and unlikely (<2.4 mg/dL) ampullary obstruction based on the level of bilirubin showed no difference between the Decrescendo and Crescendo-Decrescendo groups (Figure 2). There was no difference in age or sex between the 3 categories. The prevalence of obvious obstructive jaundice defined as bilirubin >5 mg/dL was similar in both groups (*P* = .243).

### 4.2. Common Bile Duct Obstruction

Thirty eight percent (55 of 143) of patients presented with a dilated common bile duct. Thirty-six patients (39%) in Decrescendo group had common bile duct dilatation, which was comparable to the 19 patients (41%) in the Crescendo-Decrescendo group (*P* = .76). Thirteen patients (9%) had an obvious common bile duct stone seen on ultrasound, eight patients (6%) were in the Decrescendo group, and 5 patients (2%) were in the Crescendo-Decrescendo group. There was no difference in the total duration of pain between those patients with biliary dilation and those without (*P* = .18).

ERCP was performed on 23 patients from the total population (16%). Criteria for ERCP were based on sustained rise in bilirubin levels with or without elevated white count. Significantly (*P* = .002) more patients in the Crescendo-Decrescendo group (29%, 14 of 49) underwent ERCP as compared to the Decrescendo group (10%, 9 of 94) (Table 2). The ERCP was therapeutic in 52%. No common bile duct stones were seen on ERCP in 9 (39%) patients, stone extraction was unsuccessful in 2 (9%), and sphincterotomy and/or stone extraction was performed in 12 of 23 patients (52%). There was no difference (*P* = .265) in the rate of therapeutic ERCP in the Decrescendo group (66%, 6 of 9) compared to the Crescendo-Decrescendo group (43%, 6 of 14). Three patients underwent ERCP within 48 hours of the onset of their pain. The prevalence of CBD obstruction was significantly higher (*P* = .003) in the Crescendo-Decrescendo group (31%, 15 of 49) compared to the Decrescendo group (12%, 11 of 94).

### 4.3. Bilirubin Normalization

**Figure 3:**
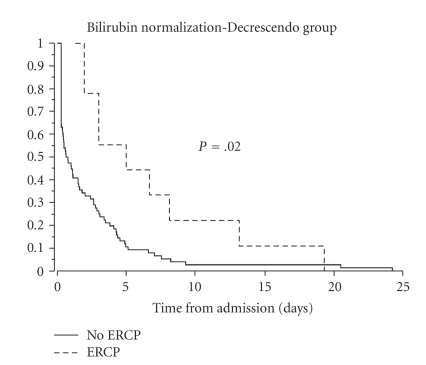
Kaplan Meier plot of Time to Normalization (<1.0 mg/dL) of bilirubin in the Decrescendo group. In patients not undergoing ERCP, time 0 was the time of admission which corresponded to the highest bilirubin level for that patient. In patients undergoing ERCP, time 0 was the day of the ERCP and time to normalization was calculated from the time of ERCP. Time to normalization in patients undergoing ERCP was significantly delayed (*P* = .02).

**Figure 4:**
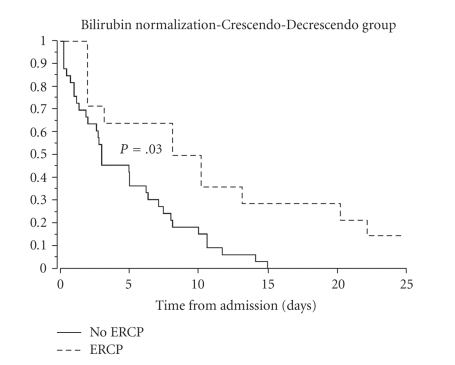
Kaplan Meier Plot of Time to Normalization (<1.0 mg/dL) of bilirubin in the Crescendo-Decrescendo group. In patients not undergoing ERCP, time 0 was the time of admission, which corresponded to the highest bilirubin level for that patient. In patients undergoing ERCP, time 0 was the day of the ERCP and time to normalization was calculated from the time of ERCP. Time to normalization in patients undergoing ERCP was significantly delayed (*P* = .03).

Median time to normalization in the Decrescendo group (21 ± 81 hours) was significantly shorter (*P* = .001) than the Crescendo group (119 ± 162 hours). If patients who underwent ERCP were excluded, time to normalization was still significantly shorter in the Decrescendo group (*P* = .001). The time to normalization of total bilirubin in patients after an ERCP was significantly longer in both groups (Figures 3 and 4).

### 4.4. Pancreatic Enzyme Levels

The majority of patients demonstrated consistent decreases in pancreatic enzymes from the time of admission. Only six (6%) patients in the Decrescendo group and 4 (8%) patients in the Crescendo-Decrescendo group had amylase and lipase levels that continued to rise after admission (*P* = .655).

## 5. Discussion

In our study we described the biochemical patterns we observed after an attack of gallstone pancreatitis, largely using bilirubin levels as a marker. Since the use of prophylactic antibiotics for severe acute pancreatitis is still a matter of discussion [13], Ranson's criteria were only used to establish the severity and to distinguish which patients will receive the antibiotics. 

It is well documented that patients with ampullary obstruction experience severe pain [14], and this presumably would be the motivating factor that brings patients to the hospital. In our study, patients with gallstone pancreatitis present at two distinct stages of the disease. The majority of patients comprise the Decrescendo group where their symptoms and their biochemical markers were on the downtrend toward recovery. These patients presented late in their disease after likely passage of the stone as evidenced by falling bilirubin levels. These patients tended to be significantly younger, and a larger proportion presented to the hospital after enduring pain for greater than 48 hours. A smaller number of patients presented in the Crescendo-Decrescendo group. These patients had escalation of their symptoms and initially increasing bilirubin levels, indicative of persistent biliary obstruction. These patients tended to be older and more presented with pain for less than 48 hours. Given the predominant indigent population that our county facility service serves it is not unexpected that the majority of our patients are young and seek medical care later in their disease process. It is possible that all patients with gallstone pancreatitis have a similar bilirubin trend after an attack of pancreatitis and that our study only categorizes patient into two groups because they were studied at or presented at two different time periods in the course of their disease. To confirm this patients would have to be studied immediately from the onset of their symptoms, which would not be practical or possible.

Studies have shown that passage of gallstones through the biliary tree is associated with pancreatitis [3, 4]. The passage is confirmed by finding of gallstones in the feces [3, 4]. Numerous studies have shown that early in the pathogenesis of gallstone pancreatitis there is obstruction of the ampulla of Vater. Acosta et al. [1, 2] and Armstrong et al. [5] demonstrated the obstruction to be mechanical with a high incidence of ampullary stones seen in their patients. Others have showed that the obstruction might also be nonmechanical and due to severe inflammation and edema of the ampulla [6, 15, 16]. Irrespective of the cause of the obstruction, our study confirmed these finding and showed that there is biochemical evidence of obstruction as depicted in the elevated serum bilirubin. 

Studies have shown that the mechanical obstruction of the biliary tract with a gallstone is usually transient and that most patients spontaneously clear their common bile duct of gallstones [1, 5]. Similarly looking at biochemical evidence of obstruction we showed that 2/3 of patients have a resolving obstruction at admission (Decrescendo group). Furthermore in this study we documented the temporal relationship of the stone passage using biochemical marker of elevated bilirubin. Only 1/3 of patients have evidence of persistent or ongoing obstruction of the biliary tract that is usually short lived with spontaneous biochemical evidence of disobstruction occurring within 2 days. Spontaneous resolution as confirmed biochemically by bilirubin normalization occurred in 90% of patients as only 10% required therapeutic intervention to remove the offending stone. This is consistent with historical papers studying gallstone pancreatitis and stone passage rates [1, 3–6]. 

An unexpected finding was the similar levels of bilirubin found at admission in both the Decrescendo group and the Crescendo-Decrescendo group of patients. Stratification of bilirubin into different levels also showed no difference between the 2 groups. Sixty percent of patients in the Crescendo-Decrescendo group had bilirubin levels on admission less than 5 mg/dL and this observation supports the finding of shorter duration of presenting symptoms in this group. The prevalence of common bile duct dilation was also similar in both groups. The patients in the Crescendo-Decrescendo group however were significantly older and combined with their earlier presentation would seem to indicate that the pain from gallstone migration or biliary tract obstruction in these patients is less well tolerated than in the younger patients. Studies have also shown that patients with gallstone pancreatitis often have repeated episode of gallstone migration down the biliary tree and have a pathological evidence of sclerosing papillitis [4]. We hypothesize that older patients may have more fibrosis of the ampulla together with decreased compliance of the common bile duct. This possibly would lead to more frequent obstruction and earlier onset of pain than in younger patients with less scarring and fibrosis, which would explain the findings in the study. Another explanation might be that in older population might have larger stones migrating through their ductal system. 

Similarly we hypothesize that endoscopic manipulation of the ampulla with or without sphincterotomy leads to inflammation and swelling of the ampulla which would explain why normalization of serum bilirubin was significantly delayed in our patient undergoing ERCP. Normalization of bilirubin levels following spontaneous disobstruction was significantly faster than normalization that occurred after ERCP. The rate of normalization was the same for both Decrescendo and Crescendo-Decrescendo groups of patients. 

An interesting observation from this study, which differs significantly from most other large series of patients with gallstone pancreatitis, was that the median age of the patients in this study was only 35 years. This is almost half the value reported by other series [8, 17–19]. Our population seems unique in consisting of a large number of young Hispanic patients and very few Medicare aged patients. This observation should be taken into account when comparing the result of this study to others. We have shown that the younger patients have a very rapid resolution of biochemical evidence of obstruction most probably related to the elasticity, increased compliance and functionality of the biliary tract that has not been subjected to repeated episodes of destructive inflammation or passage of gallstones that would be expected in older patient populations. 

Because of the high incidence of gallstones disease and gallstone pancreatitis our institution, we have adopted an aggressive approach to monitor the trend of biochemical markers with laboratory testing every 6 hours in an effort to guide the judicious use of ERCP. We have shown previously that early stratification of patients based on the likelihood of passage of stones may improve overall morbidity [15]. Those with a unremitting Crescendo pattern should perhaps considered for magnetic resonance cholangiopancreatography (MRCP) [20, 21] or ERCP. Those patients with a Decrescendo trend do not need any imaging of the CBD and can be scheduled for surgery once the pancreatitis has settled.

## Figures and Tables

**Table 1:** Patient characteristics in the Decrescendo and Crescendo-Decrescendo groups.

	Decrescendo	Crescendo-Decrescendo	*P*
	*n* = 94	*n* = 49	
Age (years)	33 (26–52)	41 (31–54)	.02
Female gender	*n* = 78 (83%)	*n* = 39 (80%)	.67
Pain <48 hours prior to Admission	47%	65%	.05
Length of stay (days)	6 (4–7.5)	6 (5–12.5)	.01

**Table 2:** Characteristics in those patients who underwent ERCP versus no ERCP.

	ERCP (*n* = 23)	No ERCP (*n* = 120)	*P*
Age (years)	41 (30–54)	34 (27–54)	.17
Duration of symptoms (hours)	48 (48–168)	48 (24–96)	.56
Female gender	16 (70%)	100 (83%)	.122
Decrescendo: Crescendo-Decrescendo group ratio	9:14	85:35	.002
CBD dilatation on ultrasound	15/23 (65%)	40/118 (34%)	.003
